# NADPH oxidase‐derived reactive oxygen species: Dosis facit venenum

**DOI:** 10.1113/EP087125

**Published:** 2019-03-07

**Authors:** Katrin Schröder

**Affiliations:** ^1^ Institute for Cardiovascular Physiology Goethe‐University Frankfurt Germany; ^2^ German Center for Cardiovascular Research (DZHK) Partner site RheinMain Frankfurt Germany

**Keywords:** NADPH oxidases, Nox2, Nox4, reactive oxygen species, redox cloud, ROS

## Abstract

**New Findings:**

- **What is the topic of this review?**
  Within this review, the role of reactive oxygen species in cellular homeostasis, physiology and pathophysiology is discussed.

- **What advances does it highlight?**
  The review provides new concepts of how reactive oxygen species influence gene expression, energy consumption and other aspects of the life of a cell. Furthermore, a model is provided to illustrate how reactive oxygen species elicit specific oxidation of target molecules.

**Abstract:**

Reactive oxygen species (ROS) have a long history of bad reputation. They are needed and effective in host defense, but on the contrary may induce situations of oxidative stress. Besides that, within recent years several soft functions (functions that may occur and are not directly connected to an effect, but may influence signaling in an indirect manner) of NADPH oxidases have been discovered, which are slowly eroding the image of the solely dangerous ROS. NADPH oxidase‐derived ROS serve to ease or enable signal transduction and to maintain homeostasis. However, there is still an enormous lag in the knowledge concerning target proteins and how ROS can elicit specific signalling in different cells and tissues. The present review summarizes some important functions of Nox2 and Nox4. Furthermore, although highly speculative, a model is provided of how those NADPH oxidases might be able to oxidize target proteins in a specific way. Many concepts mentioned in this review represent my personal view and are supported only in part by published studies.

## 1 REACTIVE OXYGEN SPECIES, NEEDED AND DANGEROUS

Reactive oxygen species (ROS) are a group of oxygen‐based, highly reactive molecules that are able to react with inert molecules, such as lipids, DNA or proteins (Buettner & Jurkiewicz, 1993). If ROS formation occurs as a side‐effect of mitochondrial dysfunction, uncoupling of enzymes, such as nitric oxide synthases (NOSs), or shifts in enzyme activity, such as in xanthine oxidase/xanthine hydroxylase, the concentration of ROS increases in an uncontrolled manner to a detrimental level, fuelling a condition called oxidative stress. The nature of oxidative stress includes unspecific oxidation of intracellular molecules, with potentially detrimental effects on cell function and survival (Misra, Sarwat, Bhakuni, Tuteja, & Tuteja, 2009). Oxidative stress often occurs in the course of unpredictable events that mainly target individual cells, such as inflammation, irradiation or poisoning, for example with cigarette smoke (Boukhenouna et al. 2018; Chen et al. 2018; Citrin & Mitchell, 2017). Although oxidative stress appears to be an accident, this is unlikely to be true. Instead, it might be that oxidative stress represents a method for self‐purification of the organism. In other words, oxidative stress and thereby cell death is not accidental; instead, it is actively provoked. Oxidative stress‐induced cell death could represent a way to get rid of poisoned or damaged cells, which makes space for new cells. Potentially, the formation of new cells is a lower cost than the repair of old or damaged ones.

## 2 NADPH OXIDASES: A DOUBLE‐EDGED SWORD OF SIGNALLING AND DAMAGE

Unlike cell‐based stress, infections with microbiota may harm the whole organism. Obviously, infections came with life of higher organisms, and nature invented a most effective defense system. Specialized cells, such as neutrophils, ingest microbiota and perform a controlled formation of ROS towards the imprisoned invader with a specialized enzyme that will kill the cell together with the invader (El‐Benna et al., 2016). This enzyme is an NADPH oxidase, whose sole function is the formation of ROS. Seven NADPH oxidases are expressed in the human body, namely Nox1–Nox5 and Duox1 and Duox2 (Brandes et al. 2014).

The NADPH oxidase involved in host defense is Nox2; an enzyme complex consisting of two membrane‐bound subunits (Nox2 and p22phox) and four cytosolic subunits (p47phox, p67phox, p40phox and Rac2). The fact that all the subunits have to assemble in order to produce superoxide anions implies that the formation of ROS by the Nox2 complex is highly controlled, and accidental activation of the complex must be prevented. This makes sense, because once activated, the Nox2 complex very rapidly produces excessive amounts of ROS, which are usually able to kill pathogenic invaders. Within the vacuole containing the pathogenic invader, myeloperoxidase converts O_2_^•^ˉ into H_2_O_2_ and HCl and other ROS, forming a toxic cocktail that will kill the invader (Rada & Leto, 2008). NADPH oxidase activation in phagocytosis also causes a pH change by proton formation that helps the proteases to digest the pathogens better. As a side note, it is important to recognize that besides killing the invader, Nox2‐derived ROS potentially also harm the cell, where it is maximally activated, in addition to surrounding cells and tissue. Additionally, Nox2‐mediated ROS formation occurs not only in response to infection, but also appears to have permanent effects, as shown for vascular reactivity (Violi et al., 2009). Permanent ‘mild’ activation of Nox2, for example, would be realized by ROS‐induced ROS formation. Mitochondrial ROS, via activation of protein kinase C, subsequently induce the phosphorylation of p47phox and thereby the assembly of the active Nox2 complex (Daiber, 2010). Vascular relaxation is dependent on NO formed by the endothelial nitric oxide synthase (eNOS). Nox2‐derived O_2_^•^ˉ reacts with NO to form ONOOˉ. This reaction not only limits the level of bioactive NO and thereby vascular relaxation (Violi et al., 2009), but also ONOOˉ potentially disturbs protein function, as too much O_2_^•^ˉ or NO would do. These issues might explain why Nox2‐derived ROS are often recognized as harmful. However, Nox2 expression is not limited to leucocytes; it is expressed in many other cells, such as endothelial cells, and the question is, why?

In fact, upon cytokine stimulation of a cell, Nox2 generates a small puff of ROS, which transiently inhibits nearby phosphatases and thereby enhances signalling (Schröder, Kohnen et al., 2009; Schröder et al., 2011). The cytokine‐induced signalling may take place even in the absence of Nox2, but in the presence of transiently activated Nox2, less effort is necessary to reach the level of intensity needed for a signal to become effective. It appears that Nox2 and, potentially, also other Nox enzymes, often serve as a switch in signalling. They enhance the walkability of existing paths, rather than opening them.

Such ‘soft skills’ of Nox2 are contrary to its function in host defense, where Nox2 is activated to the maximum. Furthermore, these soft skills are less clear and might depend strongly on the present circumstances of the cell. Therefore, they tend to be ignored and overruled by the potential harmful functions of Nox2. Many studies have been published that show a disease model with increased ROS formation, and upon treatment with antioxidants or NADPH oxidase inhibitors the disease is cured or some positive effects occur. However, it is important to recognize that the formation of ROS *per se* is not the reason for, e.g. cardiovascular diseases, and therefore reduction of ROS by antioxidants is not a cure (Hantikainen et al., 2018; Pagliaro & Penna, 2015). Although the same applies to other diseases, such as cancer or dementia (Goossens et al., 2016; Kryscio et al., 2017), the concept of ROS, NADPH oxidases and oxidative stress as the cause, and treatment with antioxidant as a potential cure of diseases, is stable, although this concept is also widely questioned (Schmidt et al., 2015; Scudellari, 2015).

## 3 DOES ENDOGENOUS ROS FORMATION BY NADPH OXIDASES REQUIRE ANTIOXIDATIVE DEFENSE?

The potential of ROS formation to have destructive effects in a healthy, unchallenged cell appears to be only minor. The lack of data that suggest a defined function or defined target molecules in the cell opens room for speculation at multiple levels. Is it really necessary for the cell to decompose ROS derived from enzymes such as NADPH oxidases? My very personal opinion is, no! Knockout of NADPH oxidases 1, 2 and 4 in mice does not result in a downregulation of ROS‐decomposing enzymes, such as superoxide dismutase or catalase (Rezende et al., 2016). Why should a cell or a mouse with no Nox1, 2 and 4 maintain the high expression of those antioxidant enzymes while the major sources of ROS are not expressed?

**Figure 1:**
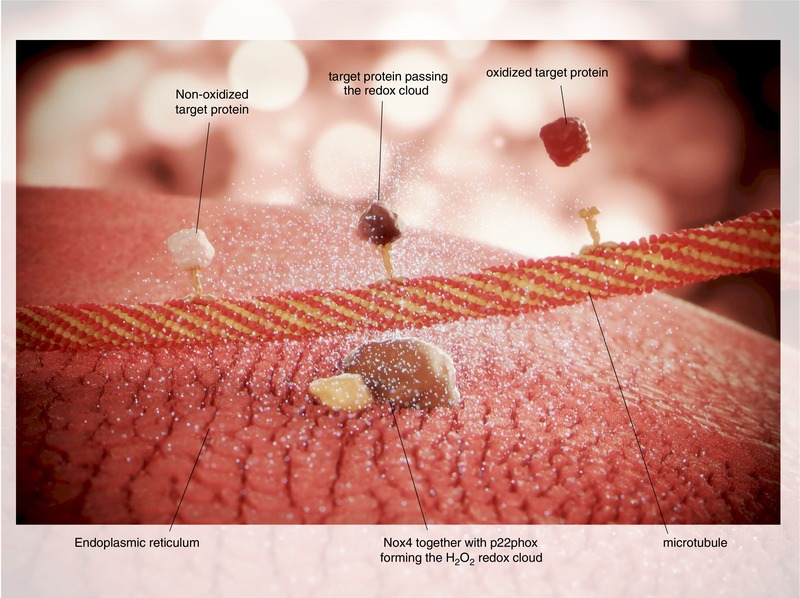
The figure shows Nox4 together with p22phox in the endoplasmatic reticulum. Nox4 produces H_2_O_2_, which builds a ‘redox cloud’. A microtubule is passing the cloud, which enables transport proteins (e.g. kinesins) to bring target proteins into the redox surrounding. This eventually results in the oxidation of the target protein and its release from the transport protein. The oxidized target protein may then mediate redox signalling, control gene expression or could even be decomposed. The possibility of oxidized transport proteins or tubulin is not shown

One speculative possibility is that local ROS formation by NADPH oxidases generates a redox cloud, which can be interpreted as a micro‐domain without a given anatomical structure (Fig. 1). Within this cloud, target molecules are oxidized, and all ROS produced are used up. In fact, it is likely that physiological ROS signalling is limited to the area in the redox cloud. One possibility is that this might be realized by clustering of ROS‐forming enzymes and target molecules (Amberg et al. 2010). Below, a new concept of localized redox signalling based on transport proteins is discussed. However, if all ROS in the redox cloud are used up, no antioxidative defense is necessary, and the absence of NADPH oxidases therefore has no effect on the expression of the antioxidative enzymes. Moreover, the expression of antioxidant enzymes could be highly conserved (regardless of the ‘real’ ROS level), because evolution taught our cells to be prepared for high ROS levels that could occur suddenly, without warning. Accordingly, the expression of antioxidative enzymes is stable. Taken together, in a healthy cell, moderate ROS formation by NADPH oxidases acts locally to oxidize target proteins, with no need for further decomposition of ROS.

In contrast, if cells or mice are treated with antioxidants, the general redox tone drops. At least in mice, supplementation of the diet with antioxidants reduced the expression of antioxidant genes (Sayin et al., 2014); Sod3 is significantly downregulated, and there is a trend towards a decrease in catalase, Sod1 and Sod2 expression (Clotilde Wiel and Martin Bergo, personal communication). In conclusion, dietary supplementation with antioxidants reduces the ability of the cell to resist a situation of oxidative stress. In fact, moderate oxidative stress probably represents a preconditioning mechanism to prepare the cell for future more severe damage (e.g. as the induction of Nrf2 and downstream protective genes; Cuadrado et al., 2018). Accordingly, expression of the antioxidative enzymes decreases in situations with a general reduction of the redox tone. How does a cell then sense this drop and why do antioxidants reduce the expression of superoxide dismutase (SOD) etc.? Following the above‐mentioned speculation, general reduction of all ROS disturbs the formation of redox clouds by any source (e.g. NADPH oxidases and mitochondria) and thereby prevents oxidation of target proteins. Among those target genes, one or several may serve as redox sensors. If oxidized, those redox sensors will activate the expression of antioxidative genes. A known redox‐dependent element that controls SOD expression is the antioxidative response element, the activation of which increases the expression of Sod3 and Activator protein 1 (AP1), which suppresses the expression of Sod2 (Zelko et al. 2002).

These data indicate the existence of a feedback mechanism that controls the general level of ROS and antioxidative genes. Taken together, the findings suggest that a certain level or redox tone is part of cellular homeostasis.

## 4 NOX4‐DERIVED H_2_O_2_ IS ESSENTIAL TO MAINTAIN CELLULAR HOMEOSTASIS

The redox tone of a cell is, in fact, a major component of homeostasis (Ursini et al. 2016). The NADPH oxidase Nox4 represents an important source of ROS. This specific NADPH oxidase was first found to be expressed in the kidney, although its function in that organ remains to be discovered. Meanwhile, it is clear that basically every cell expresses Nox4 (The Tabula Moris Consortium, 2018). Several effects of Nox4 have been published, and most of them belong to the ‘soft skills’ mentioned before. Especially in the case of Nox4, the positive correlation between the lack of knowledge of the real enzyme function and the number of studies following the approved pattern to show harmful effects of the enzyme and the cure by antioxidants is obvious.

Unlike most other members of the NADPH oxidase family, Nox4 is constitutively active. This means that the cell can balance the demand for ROS and its production by controlling the expression of Nox4. Importantly, Nox4 produces H_2_O_2_ (Helmcke, Heumüller, Tikkanen, Schröder, & Brandes, 2009). Unlike superoxide anions, H_2_O_2_ can directly oxidize proteins at cysteine or methionine residues, and therefore no further transmitters or signal chains are needed. This means that if the redox cloud model applies, intracellular localization of proteins determines their oxidation status. Further speculation suggests that the transport rate of proteins towards and out of the cloud, and therefore transport proteins (e.g. kinesins), might be major determinants of which proteins are oxidized, and how many and to what degree. Nox4 mediates a permanent redox signal, which enables long‐term processes, such as differentiation (Goettsch et al., 2013; Schröder, Wandzioch et al. 2009) and cellular quiescence (Schröder et al., 2012). This system might apply as long as no extraordinary increase or decrease in ROS formation occurs. In the case of no ROS formation or too little, inefficient oxidation of the proteins takes place, which may be recognized by the transport system. Consequently, the transport rates increase but remain without any effect. This eventually exhausts and devitalizes the cell, making it more susceptible to challenges by external stressors. Consequently, in healthy cells, such as isolated lung endothelial cells, the loss of Nox4 promotes apoptosis (Schröder et al., 2012). In turn, little cell stress increases the expression of Nox4 (Babelova et al., 2012; Lee et al. 2013), thereby ROS formation escalates and makes the redox modification more efficient. Especially in the case of Nox4, it appears that more efficient oxidation has protective effects, at least in the heart, where overexpression of Nox4 prevents cardiac remodelling upon pressure overload (Zhang et al., 2010). Nox4, in fact, might not be necessary to obtain cellular homeostasis, but it appears to be necessary to maintain it.

The diverse role of Nox4 is illustrated in the setting of cancer. In a healthy cell, Nox4 maintains genomic stability and controls proliferative activity, e.g. via oxidation of targets such as Akt. Nox4 prevents inflammatory activation and dedifferentiation of cells. Accordingly, knockout of *Nox4* promotes the development of solid tumors in pro‐inflammatory mouse‐models for cancer (Helfinger et al. 2017). In contrast, in existing cancers Nox4 is highly upregulated (https://www.proteinatlas.org/ENSG00000086991-NOX4/pathology). This upregulation, however, is not necessarily associated with lower survival of the patient; in fact, in renal cancer a high expression of Nox4 prolongs the survival of the patient (https://www.proteinatlas.org/ENSG00000086991-NOX4/pathology/tissue/renal+cancer). In cell culture, in most cases upregulation of Nox4 promotes survival and prevents apoptosis, e.g. of ECV304 cancer cells (Giannoni et al., 2008).

Going back to the above‐mentioned redox cloud model, the upregulation of Nox4 in cancer cells eases oxidation of target proteins and reduces the effort needed for their ‘cloud transportation’. Importantly, a cancer cell is usually at the limit of its metabolic possibilities (Romero‐Garcia, Lopez‐Gonzalez, Báez‐Viveros, Aguilar‐Cazares, & Prado‐Garcia, 2011). Therefore, any enhancement of demands will be detrimental for the cell, as shown for many cancers. According to the redox cloud model, reduction or destruction of the cloud will increase the rate of transport of intracellular proteins, in order to get them oxidized. However, this remains ineffective, and the cell is easily exhausted. Inhibition of Nox4, therefore, might prevent the survival of an existing cancer cell. It is possible that this is not a specific effect of Nox4, because general inhibition of ROS formation using DPI or specific downregulation of Nox2, at least in osteosarcoma cells, also promotes apoptosis (Kitamoto et al., 2018). Recent studies, in fact, show positive outcomes if cancer is treated with high concentrations of the antioxidant vitamin C (Schoenfeld et al., 2017).

## 5 CONCLUDING REMARKS

NADPH oxidases are a group of enzymes whose sole function is to produce ROS. In situations of overwhelming ROS formation, as occurs in inflammation and host defense, cells may be damaged. In contrast, NADPH oxidases provide several ‘soft skills’. For instance, they may ease signal transduction by transient inhibition of phosphatases. Furthermore, they contribute to cell homeostasis. Although ROS may not be able to recognize any specific target protein, it is likely that they form redox clouds and that transport proteins specify which target proteins are oxidized. Although highly speculative, this scenario might explain how ROS elicit specific signalling.

## COMPETING INTERESTS

None declared.
